# One-Dimensional Photonic Crystals with Nonbranched Pores Prepared via Phosphorous Acid Anodizing of Aluminium

**DOI:** 10.3390/nano12091548

**Published:** 2022-05-03

**Authors:** Sergey E. Kushnir, Nina A. Sapoletova, Ilya V. Roslyakov, Kirill S. Napolskii

**Affiliations:** 1 Department of Chemistry, Lomonosov Moscow State University, Moscow 119991, Russia; nina@elch.chem.msu.ru (N.A.S.); kirill@inorg.chem.msu.ru (K.S.N.); 2 Department of Materials Science, Lomonosov Moscow State University, Moscow 119991, Russia; ilya.roslyakov@gmail.com

**Keywords:** anodic aluminium oxide, photonic crystal, phosphorous acid, anodizing

## Abstract

One-dimensional photonic crystals (1D PhCs) obtained by aluminium anodizing under oscillating conditions are promising materials with structure-dependent optical properties. Electrolytes based on sulphuric, oxalic, and selenic acids have been utilized for the preparation of anodic aluminium oxide (AAO) 1D PhCs with sub-100-nm pore diameter. AAO films with larger pores can be obtained by anodizing in phosphorous acid at high voltages. Here, for the first time, anodizing in phosphorous acid is applied for the preparation of AAO 1D PhCs with nonbranched macropores. The sine wave profile of anodizing voltage in the 135–165 V range produces straight pores, whose diameter is above 100 nm and alternates periodically in size. The pore diameter modulation period linearly increases with the charge density by a factor of 599 ± 15 nm·cm^2^·C^−1^. The position of the photonic band gap is controlled precisely in the 0.63–1.96 µm range, and the effective refractive index of AAO 1D PhCs is 1.58 ± 0.05.

## 1. Introduction

Photonic crystals (PhCs) are structured materials that possess photonic band gaps—wavelength bands in the optical range, where light reflects on the periodic structure due to Bragg diffraction [1,2,3]. Valve metals’ anodizing under periodically oscillating conditions is a low-cost, scalable, and reproducible method of preparing one-dimensional (1D) PhCs [4,5,6,7,8,9,10]. PhCs based on anodic aluminium oxide (AAO) are used in chemical sensors [5,11,12,13,14], low-threshold lasers [15,16], optical filters [17,18], photonic tags [19], and photocatalysis [20,21].

Electrolytes based on sulphuric [5,8,22,23,24,25], oxalic [4,26,27,28], and selenic [29] acids have been utilized for the preparation of AAO 1D PhCs with sub-100-nm pore diameter (Table S1). AAO films with larger pores can be obtained by anodizing in phosphorous acid at high voltages [30]. To the best of our knowledge, the synthesis of 1D PhCs in the phosphorous acid bath and their optical properties have not been reported yet.

Recently, an anodizing regime with voltage (*U*) versus electric charge (*Q*) modulation, *U*(*Q*), was successfully used to prepare anodic titanium oxide PhCs with precisely controlled morphology [31,32]. In situ measuring of *Q* allows one to control the thickness of the formed layers with high precision, whereas control of *U* guarantees the identity of the voltage-dependent parameters of the porous structure for each layer from the top to the bottom. In the case of AAO, the porous film thickness linearly increases with the charge density (*q*) spent during anodizing. The proportionality coefficient depends on anodizing conditions (electrolyte composition, voltages) and lies in the range of 360–630 nm·cm^2^·C^–1^ [8,24,29,33,34,35,36,37,38]. Here, for the first time the *U*(*Q*) anodizing regime with a sine-wave voltage profile is applied in the phosphorous acid electrolyte to prepare AAO 1D PhCs. Scanning electron microscopy and optical spectroscopy measurements are used to characterize the morphology and optical properties of the PhCs. It is demonstrated that AAO 1D PhCs maintain their properties at temperature up to 100 °C.

## 2. Materials and Methods

H_3_PO_3_ (99%), H_3_PO_4_ (85% aqueous solution), CrO_3_ (99.7%), Br_2_ (98%), and CH_3_OH (99.9%) were used as received, i.e., without further purification steps. All aqueous solutions were prepared with distilled water.

**Figure 1:**
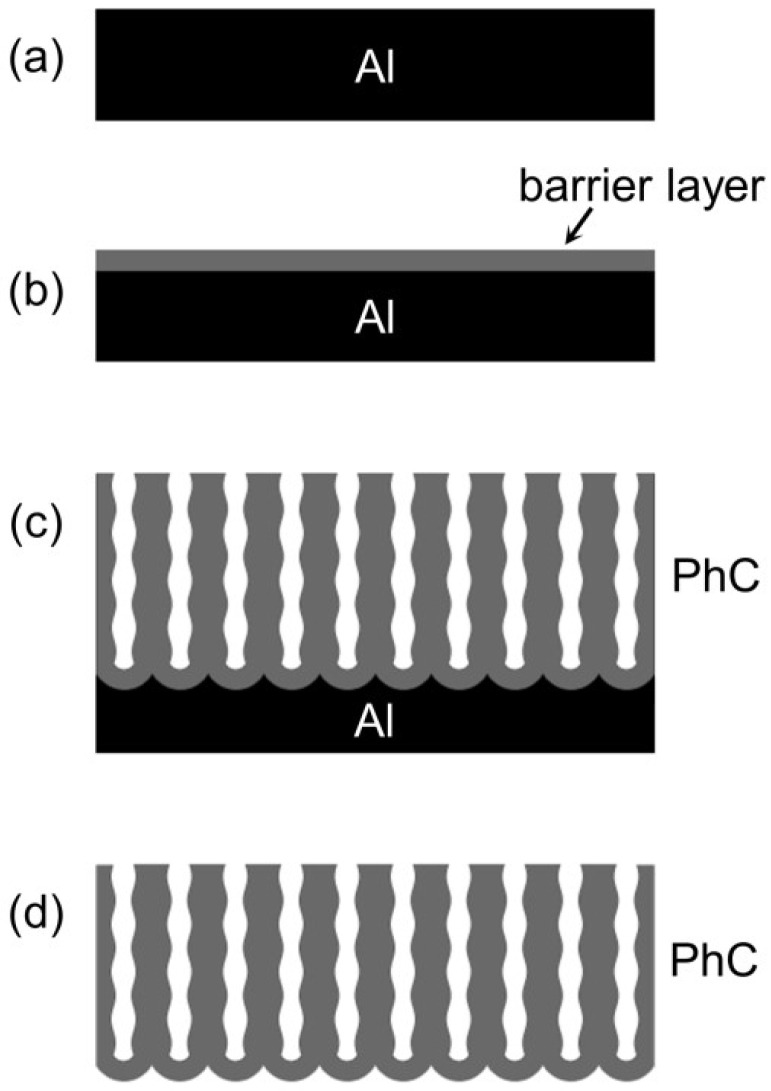
Synthesis of anodic aluminium oxide (AAO) one-dimensional photonic crystal (1D PhC). (**a**) Electropolished aluminium foil. (**b**) Barrier-type anodic alumina layer grown on the Al foil. (**c**) 1D PhC prepared by aluminium anodizing in phosphorous acid electrolyte. (**d**) Free-standing 1D PhC.

High-purity aluminium foils (99.99%, 0.1 mm thick) were electrochemically polished to a mirror finish (Figure 1a) in a solution containing 12.85 M H_3_PO_4_ and 1.85 M CrO_3_ at 80 °C, as described elsewhere [24,39]. Polishing was carried out in impulse mode. The foil was polarized 40 times for 3 s at an anodic current density of 0.5 A·cm^−2^. The delay time between pulses was 40 s.

The preparation of AAO porous films was performed in a two-electrode electrochemical cell with an Al cathode. The electrolyte was agitated at a rate of 480 rpm using an overhead stirrer. The electrolyte was maintained at constant temperature during the anodizing using a Huber CC-K6 cryostat. Firstly (Figure 1b), an oxide barrier layer was formed on the aluminium surface by anodizing in 0.1 M H_3_PO_4_ at 150 V for 30 s and the electrolyte temperature of 0 ± 0.1 °C. The barrier layer allows one to avoid AAO burning during the formation of PhCs. Further anodizing in 1 M H_3_PO_3_ (Figure 1c) was performed on the anodizing area of 0.37 ± 0.01 cm^2^ (Figure S1) at 4.5 ± 0.1 °C. Voltage, as a function of charge passed during anodizing, was applied using a programmable DC power supply Agilent N5771A, as described previously [31]. Briefly, the anodizing voltage was set according to predesigned *U*(*Q*) profile, where *Q* was calculated by integrating the current that was measured every ~110 ms. The sine wave *U*(*Q*) profile with *U* in the 135–165 V range was used to prepare AAO PhCs:

(1) $$U\left(Q\right)=150+15\cdot \sin\left(2\pi \frac{Q}{Q_{0}}\right),$$

where *Q*_0_ is the period of the *U*(*Q*) profile, which determines the periodicity of porosity modulation along the normal to the AAO film. The total charge was the same for all the samples, whereas the number of cycles was dependent on *Q*_0_. Samples S1, S2, S3, S4, S5, and S6 were obtained at the following parameters of charge density per cycle (*q*_0_) and the number of anodizing cycles (*N*): 0.330 C·cm^−2^ × 130, 0.418 C·cm^−2^ × 100, 0.534 C·cm^−2^ × 80, 0.632 C·cm^−2^ × 65, 0.832 C·cm^−2^ × 50, and 1.043 C·cm^−2^ × 40, respectively. After anodizing, the AAO films were washed with water and then dried in air. The residual Al was selectively dissolved using the 9 vol.% bromine solution in methanol (Figure 1d).

The optical properties of the samples were analysed using a Lambda 950 spectrophotometer (PerkinElmer, Waltham, MA, USA). The transmittance spectra of the PhCs were collected in the 200 to 2500 nm range. A step of 2 nm and a slit width of 2 nm were used to collect specular transmittance spectra, whereas a step of 5 nm and a slit width of 5 nm were used to collect total transmittance spectra.

The morphology of the AAO films was characterized using a scanning electron microscope (SEM) Leo Supra 50VP (Carl Zeiss SMT, Oberkochen, Germany). Before SEM investigations, the samples were covered with a 7-nm-thick conductive Cr layer using a Q150T ES sputter coater (Quorum Technologies, Laughton, East Sussex, United Kingdom). To analyse the interpore distance distribution, SEM images of a barrier layer were statistically analysed using the ImageJ software (version 1.49v, Wayne Rasband, National Institutes of Health, USA) [40] and a lab-made Statistics2D program (version 1.4, Dmitry S. Koshkodaev, Moscow State University, Russia) [41].

## 3. Results and Discussion

**Figure 2:**
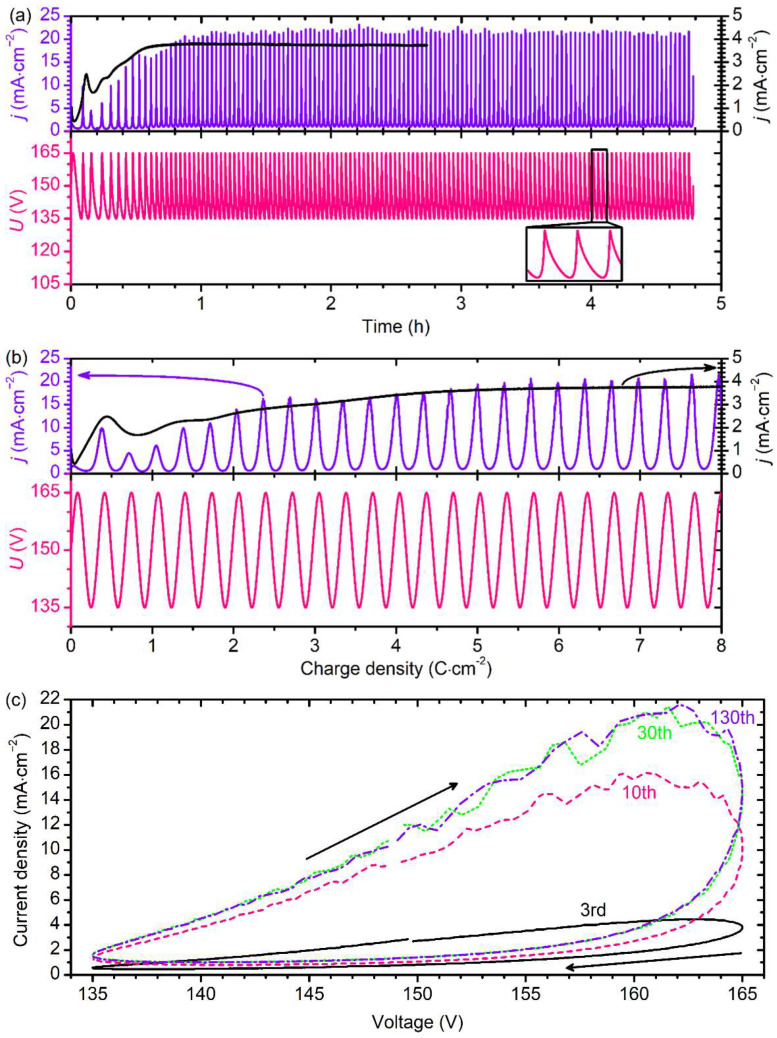
Electrochemical responses recorded during aluminium anodizing in 1 M H_3_PO_3_ at 4.5 °C under sine wave voltage versus electric charge modulation in the 135–165 V range. Data for the sample S1 (0.330 C·cm^−2^ × 130) are shown. (**a**) Time dependences of anodizing voltage (*U*) and current density (*j*). (**b**) Dependences of *U* and measured *j* values on charge density. The black curve represents the current density in the case of potentiostatic anodizing at 150 V. (**c**) *j*-*U* plots for various anodizing cycles: 3rd (solid black line), 10th (dashed pink line), 30th (dotted green line), and 130th (dashed-dotted violet line).

Figure 2 shows the typical electrochemical responses recorded during the formation of AAO PhCs in 1 M H_3_PO_3_. Sine wave modulation of the anodizing voltage versus charge density results in an oscillation of the current density (*j*). During the first 24 cycles (up to *q* = 8 C·cm^−2^), the maximum and minimum values of *j* were nonmonotonous (Figure 2b). Later, from the 25th to 130th cycles, the values of *j* were nearly the same from cycle to cycle (Figure 2a). More than five-fold growth in AAO thickness resulted in just a slight decrease in *j*. The observed behaviour indicates that the AAO films were formed in a kinetic regime [42] in which *j* does not depend on the AAO thickness. In the case of potentiostatic anodizing at 150 V, *j* as a function of *q* behaved similarly to an envelope of *j* maxima observed during sine wave modulation (Figure 2b). It can be clearly seen that the increase in *U* from 135 to 165 V took less time than the *U* decrease (see inset in Figure 2a); this behaviour is caused by the hysteresis loop on the *j*(*U*) curve (Figure 2c). For each anodizing cycle, *j* was higher on the half-period of the *U*(*Q*) sine wave when *U* increased. Note that the observed curves were qualitatively the same for all the obtained PhCs.

**Figure 3:**
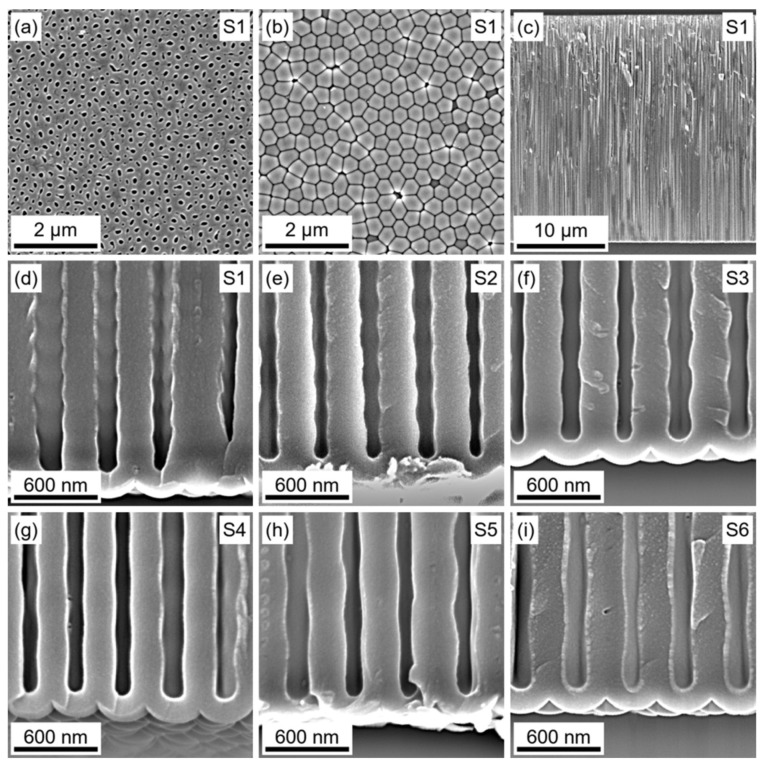
Morphology of AAO 1D PhCs prepared in 1 M H_3_PO_3_ at 4.5 °C under sine wave voltage versus electric charge modulation in the 135–165 V range. Scanning electron microscope (SEM) images of the sample S1 (0.330 C·cm^−2^ × 130): the top (**a**) and bottom (**b**) surfaces of the AAO film, and cleavage of the sample (**c**). Enlarged views of the cleavages of the samples S1–S6 with various charge densities per cycle (*q*_0_) and the number of anodizing cycles (*N*): 0.330 C·cm^−2^ × 130 (**d**), 0.418 C·cm^−2^ × 100 (**e**), 0.534 C·cm^−2^ × 80 (**f**), 0.632 C·cm^−2^ × 65 (**g**), 0.832 C·cm^−2^ × 50 (**h**), and 1.043 C·cm^−2^ × 40 (**i**).

According to [43], a *U* decrease of √2 times is required for the splitting of each pore into two smaller ones. In the present study, the ratio of the highest to the lowest values of *U* was 1.22, which was lower than √2. Thus, an AAO morphology with straight nonbranched pores could be expected. Indeed, vertically aligned pores with alternating diameters along the normal to the film and without branching was clearly seen (Figure 3). The observed morphology was not typical for the 1D PhCs obtained by aluminium anodizing in sulphuric [8,24], selenic [29], and oxalic [26,44] acids, when a voltage modulation greater than √2 was applied (Table S1).

The pore diameter in the obtained AAO films alternated in a similar manner as the inner tube diameter in the anodic titanium oxide PhCs obtained using the *U*(*Q*) anodizing regime [31,32]. The thickness–to–charge density ratio, calculated by dividing the AAO film thickness by the charge density passed during anodizing, was 599 ± 15 nm·cm^2^·C^−1^. The pore diameter was estimated according to the SEM images of the AAO film cleavages (Figure 3d–i). Clearly, the apparent pore diameter in Figure 3d–i underestimated the intrinsic one, because the fracture did not occur precisely through the pore centre. However, the highest diameter value in the population of the pores was close to the intrinsic one. According to the analysis of SEM images, the pore diameter alternated between 135 and 170 nm. This range of pore diameter was unreachable via aluminium anodizing in sulphuric, selenic, and oxalic acid electrolytes. The period of the pore diameter modulation linearly increased with *q*_0_ from 208 to 633 nm.

**Figure 4:**
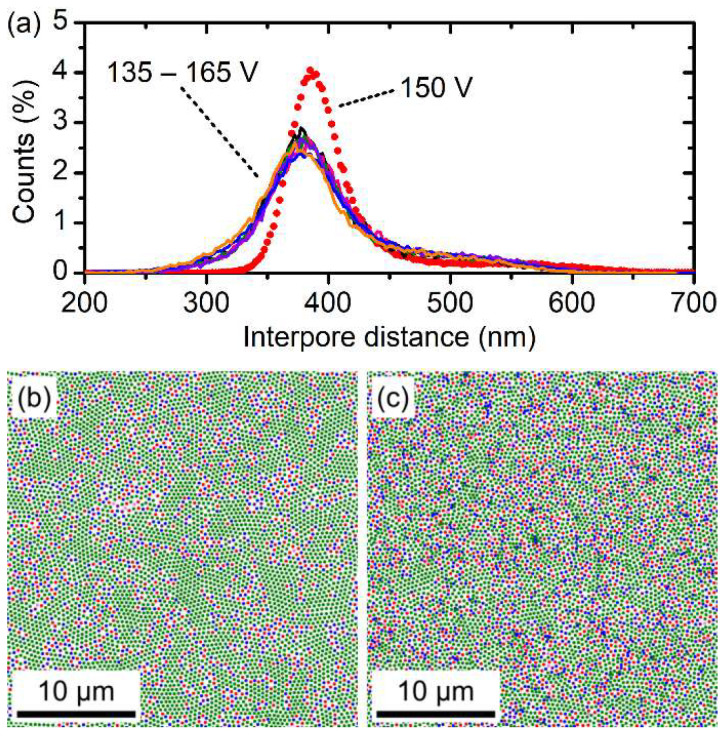
Statistical analysis of SEM data. (**a**) Interpore distance distributions obtained for the AAO films prepared at the constant voltage of 150 V (red dots) and at the modulated voltage in the 135–165 V range with various *q*_0_ × *N*: 0.330 C·cm^−2^ × 130 (black), 0.418 C·cm^−2^ × 100 (pink), 0.534 C·cm^−2^ × 80 (green), 0.632 C·cm^−2^ × 65 (violet), 0.832 C·cm^−2^ × 50 (blue), and 1.043 C·cm^−2^ × 40 (orange). Colour-coded maps for the AAO films prepared at the constant voltage of 150 V (**b**) and at the modulated voltage in the 135–165 V range with *q*_0_ × *N* of 0.632 C·cm^−2^ × 65 (**c**). Colours in panels (**b**,**c**) indicate the number of nearest neighbours of the considered pore: four—pink, five—red, six—green, seven—blue, and eight—violet.

The interpore distance (*D*_int_) distribution diagrams obtained by statistical analysis of the bottom-view SEM images of the AAO films (Figure 3b) are shown in Figure 4. It can be clearly seen that the average *D*_int_ observed for the film formed during the sine wave modulation of *U* in the 135–165 V range was slightly lower than the *D*_int_ for the sample obtained at 150 V (the average value between 135 and 165 V). Furthermore, colour-coded maps in Figure 4b,c show that the sample obtained at 150 V demonstrated larger areas with a hexagonal pore arrangement (green dots). In contrast, the porous structure of PhCs consisted of many pores with five and seven nearest neighbours. Statistical analysis revealed 71% of pores in hexagonal coordination in the case of the sample obtained at *U* = 150 V, whereas PhCs possessed only 58–60% of pores in hexagonal coordination.

**Figure 5:**
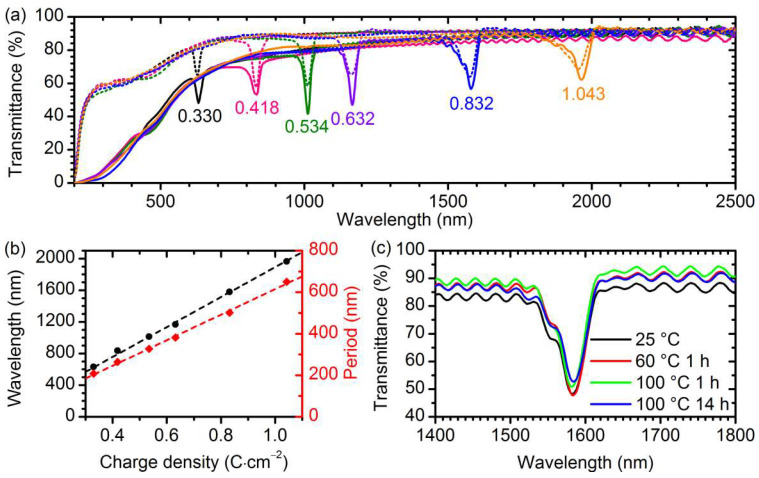
Optical properties of AAO 1D PhCs prepared by aluminium anodizing in 1 M H_3_PO_3_. (**a**) Specular (solid lines) and total (dotted lines) transmittance spectra of the samples with various *q*_0_ × *N*: 0.330 C·cm^−2^ × 130 (black), 0.418 C·cm^−2^ × 100 (pink), 0.534 C·cm^−2^ × 80 (green), 0.632 C·cm^−2^ × 65 (violet), 0.832 C·cm^−2^ × 50 (blue), and 1.043 C·cm^−2^ × 40 (orange). The transmittance minima are labelled with the values of *q*_0_ (C·cm^−2^). (**b**) Dependence of the wavelength position of the photonic band gap and the period of AAO 1D PhCs on the charge density consumed for one anodizing cycle. The dashed lines represent the linear fitting of the experimental data. (**c**) Specular transmittance spectra of the sample 0.832 C·cm^−2^ × 50 after successive aging for 2 months at 25 °C (black), 1 h at 60 °C (red), 1 h at 100 °C (green), and 14 h at 100 °C (blue).

Figure 5a shows the transmittance spectra of the prepared AAO PhCs. The photonic band gaps are clearly seen as transmittance minima, whose positions shifted to higher wavelengths with the growth in *q*_0_. The area of the transmittance peak within the photonic band gap increased with wavelength, which could be caused by the decrease in the light scattering with the growth of the wavelength–to–pore diameter ratio [45,46]. The specular and total transmittance outside the photonic band gap grew with the wavelength as well and exceeded 73% and 87%, respectively, in the near-IR range for all the samples. The position of the photonic band gap increased linearly with *q*_0_ with the slope of 1892 ± 15 nm·cm^2^·C^−1^ (Figure 5b). According to the Bragg–Snell law [47], the position of the first photonic band gap (PBG) is:

(2) $$\lambda =2d\sqrt{n_{\mathrm{eff}}^{2}-\sin^{2}\theta },$$

where *λ* is the wavelength of the first PBG, *d* is the structure period, *n*_eff_ is the effective refractive of the AAO PhC, and *θ* is the angle of incidence. In the case of the normal incidence (*θ* = 0°), *n*_eff_ = *λ*/(2*d*). For the prepared PhCs, *n*_eff_ was 1.58 ± 0.05. The estimated value of total reflectance from the PhC sides, caused by the difference in *n*_eff_ and the refractive index of air [48], was 9.5% (see Supplementary Materials “Estimation of total reflectance” and Figure S2 for more details). Thus, the absorbance inside the AAO PhCs was, evidently, below 4% in the near-IR range.

The PBG width (in frequency space, Δ*f*) of a model periodic multilayer structure consisting of multiple double layers of the same optical thickness with refractive indices *n*_a_ and *n*_b_ (*n*_b_ > *n*_a_) at normal incidence of light is as follows [49]:

(3) $$\Delta f\approx \frac{8c}{\lambda }\frac{n_{b}-n_{a}}{n_{b}+n_{a}},$$

where *c* is the speed of light. In the case of *n*_b_ − *n*_a_ << *n*_b_ + *n*_a_, the PBG width (in wavelength units, Δ*λ*) is described by following equation:

(4) $$\frac{\Delta \lambda }{\lambda }\approx 4\frac{\Delta n}{n_{\mathrm{eff}}},$$

where Δ*n* is the refractive index contrast (*n*_b_−*n*_a_)_._

Equations (3) and (4) show that the PBG width increased with PBG position and the refractive index contrast. Δ*λ* of the prepared AAO PhCs lied in the range of 23–61 nm, whereas Δ*λ/λ* was in the range of 0.023–0.037. It should be noted that the AAO PhCs differed from the model layered structure by: (i) the smooth variation of the refractive index between *n*_a_ and *n*_b_; (ii) the imperfections of the PhC microstructure, e.g., the dispersion of the optical period of the structure due to pore widening during anodizing. Thus, Equation (3) provides only a rough estimate of Δ*n* ≈ 0.01.

It is worth noting that the optical characteristics of the samples remained constant during long-term aging. The PBG position of the prepared sample S5 was 1580 nm and deviated by less than 4 nm (Figure 5c) after successive aging for 2 months at 25 °C (1582 nm), 1 h at 60 °C (1584 nm), 1 h at 100 °C (1582 nm), and 14 h at 100 °C (1584 nm).

## 4. Conclusions

Phosphorous acid anodizing was successfully used for the preparation of one-dimensional anodic aluminium oxide photonic crystals for the first time. Aluminium cyclic anodizing in 1.0 M H_3_PO_3_ at 4.5 °C under sine wave *U*(*Q*) modulation in the range of 135–165 V resulted in the formation of 1D photonic crystals. Varying the electric charge density consumed for one cycle of anodizing from 0.33 to 1.04 C·cm^−2^ allowed one to tune the position of the photonic band gap in a range from 0.63 to 1.96 μm. Under the used conditions, straight nonbranched pores with alternative diameters above 100 nm were formed; the thickness–to–charge density ratio was 599 ± 15 nm·cm^2^·C^–1^. The effective refractive index of the obtained photonic crystals was 1.58 ± 0.05.

## Data Availability

The data presented in this study are available in the article.

## Supplementary Materials

The following supporting information can be downloaded at: https://www.mdpi.com/article/10.3390/nano12091548/s1, Table S1: Preparation conditions and parameters of morphology of anodic aluminium oxide one-dimensional photonic crystals; Figure S1: Scanned images of the S1–S6 samples; Estimation of total reflectance; Figure S2: Total reflectance spectra of the S1–S6 samples.
