# Supplementary material for: One-Dimensional Photonic Crystals with Nonbranched Pores Prepared via Phosphorous Acid Anodizing of Aluminium
Source: Nanomaterials (Basel). 2022 May 3;12(9):1548. doi: 10.3390/nano12091548 (PMC9103521; doi:10.3390/nano12091548)
Supplement: Supplementary file 1 [file nanomaterials-12-01548-s001.zip › nanomaterials-1685375-supplementary.pdf]

Supplementary Material

One-Dimensional Photonic Crystals with Nonbranched Pores Prepared via Phosphorous Acid Anodizing of Aluminium

Sergey E. Kushnir [1,2,*], Nina A. Sapoletova [1], Ilya V. Roslyakov [2] and Kirill S. Napolskii [1,2]

1 Department of Chemistry, Lomonosov Moscow State University, Moscow 119991, Russia; nina@elch.chem.msu.ru (N.A.S.); kirill@inorg.chem.msu.ru (K.S.N.)
2 Department of Materials Science, Lomonosov Moscow State University, Moscow 119991, Russia;
ilya.roslyakov@gmail.com
* Correspondence: kushnir@elch.chem.msu.ru

Table S1. Preparation conditions and parameters of morphology of anodic aluminium oxide one-dimensional photonic crystals.

**Electrolyte**		Anodizing regime	Voltage range, V	Number of periods	D_p, nm	D_{int}, nm	Pore type	Reference
**H_2SO_4**	0.3 M	$j(t)$		30–90	< 25		branched pores	[1]
	1 M		6.7–22	25–300	< 25			[2]
	1.1 M		6.6–15.8	145	< 40			[3]
	1.1 M + 25 vol. % EtOH		6–16.5	62–310	6–22			[4]
	2 M	$U(L)$	10–15	100	< 20	25–38		[5,6]
H_2SeO_4	1 M	$j(t)$	14.4-33.3	75	< 17	40.5–64.7		[7]
$H_2C_2O_4$	0.3 M	$U(t)$	23–53	~170	40–80	~ 100	branched pores before chemical etching	[8]
			23–58.4	100	< 65		branched pores	[9]
			23–53	400	35–82			[10]
		$j(t)$		200	32–44	103–108		[11]
H_3PO_3	1 M	$U(Q)$	135–165	40–130	135–170	380	non-branched pores	Present work

j – current density, t – time, U – voltage, L – optical path length, Q – electric charge, D_p – pore diameter, D_{int} – interpore distance, EtOH – ethanol.

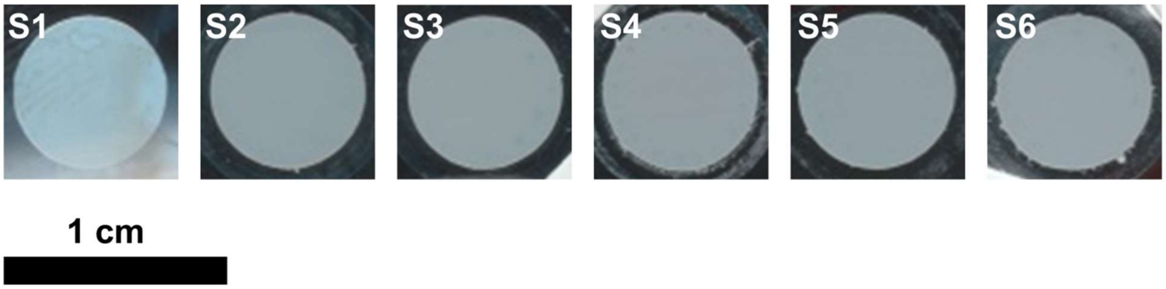

Figure S1. Scanned images of AAO 1D PhCs. The samples names are shown in the upper left corner of the images.

Estimation of total reflectance

At normal incidence, the reflectance (R) of the boundary between air and a dielectric with the refractive index n can be calculated as [12]:

$$R = \left(\frac{n-1}{n+1}\right)^2. \quad (1)$$

For the prepared PhCs, n_{eff} is 1.58 ± 0.05. According to Eq. (1) the front side reflectance of PhC is 5%. The back side of PhC also reflects 5% of the incident light. However, the intensity of the incident light on the back side of PhC is <100%. Considering the measured intensity of transmitted light of about 90%, the reflectance from the back side can be estimated as 4.5%. The reflectance from both sides, caused by the difference in n_{eff} and the refractive index of air, gives the estimated value of 9.5%. Note that the interference of the reflected light results in the Fabry–Pérot fringes with reflectance depending on the wavelength: higher or lower than estimated.

Another estimation of the total reflectance of the PhCs at the normal incidence can be extracted from the total reflectance spectra of the prepared PhCs at the incidence angle of 8° using Spectralon diffuse reflectance standard (Figure S2). Most probably, high values of the total reflectance at the wavelengths below 700 nm are caused by the light backscattering inside the PhCs.

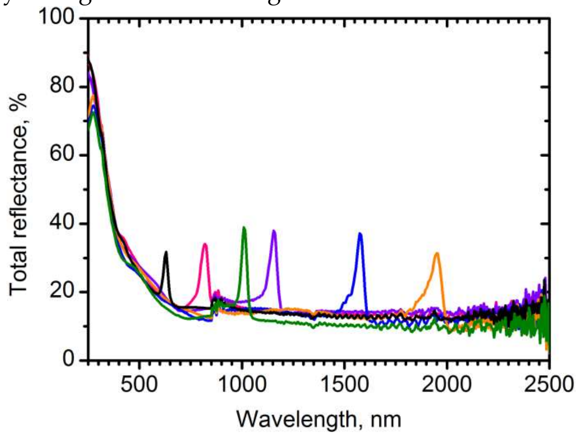

Figure S2. Total reflectance spectra of the AAO 1D PhCs with various $q_0 \times N$: 0.330 C·cm^{-2} × 130 (black), 0.418 C·cm^{-2} × 100 (pink), 0.534 C·cm^{-2} × 80 (green), 0.632 C·cm^{-2} × 65 (violet), 0.832 C·cm^{-2} × 50 (blue), and 1.043 C·cm^{-2} × 40 (orange). The spectra are recorded at an incident angle of 8°.

References

1. Guo, D.-L.; Fan, L.-X.; Wang, F.-H.; Huang, S.-Y.; Zou, X.-W. Porous Anodic Aluminum Oxide Bragg Stacks as Chemical Sensors. *J. Phys. Chem. C* **2008**, *112*, 17952–17956, doi:10.1021/jp806926f.
2. Kushnir, S.E.; Napolskii, K.S. Thickness-Dependent Iridescence of One-Dimensional Photonic Crystals Based on Anodic Alumina. *Mater. Des.* **2018**, *144*, 140–150, doi:10.1016/j.matdes.2018.02.012.

3. Liu, Y.; Chang, Y.; Ling, Z.; Hu, X.; Li, Y. Structural Coloring of Aluminum. *Electrochem. Commun.* **2011**, *13*, 1336–1339, doi:10.1016/j.elecom.2011.08.008.
4. Law, C.S.; Santos, A.; Nemati, M.; Losic, D. Structural Engineering of Nanoporous Anodic Alumina Photonic Crystals by Sawtooth-like Pulse Anodization. *ACS Appl. Mater. Interfaces* **2016**, *8*, 13542–13554, doi:10.1021/acsami.6b03900.
5. Kushnir, S.E.; Pchelyakova, T.Yu.; Napolskii, K.S. Anodizing with Voltage versus Optical Path Length Modulation: A New Tool for the Preparation of Photonic Structures. *J. Mater. Chem. C* **2018**, *6*, 12192–12199, doi:10.1039/C8TC04246B.
6. Kushnir, S.E.; Komarova, T.Yu.; Napolskii, K.S. High-Quality-Factor Anodic Alumina Optical Microcavities Prepared by Cyclic Anodizing with Voltage versus Optical Path Length Modulation. *J. Mater. Chem. C* **2020**, *8*, 3991–3995, doi:10.1039/C9TC07079F.
7. Sadykov, A.I.; Kushnir, S.E.; Roslyakov, I.V.; Baranchikov, A.E.; Napolskii, K.S. Selenic Acid Anodizing of Aluminium for Preparation of 1D Photonic Crystals. *Electrochem. Commun.* **2019**, *100*, 104–107, doi:10.1016/j.elecom.2019.01.027.
8. Wang, B.; Fei, G.T.; Wang, M.; Kong, M.G.; Zhang, L.D. Preparation of Photonic Crystals Made of Air Pores in Anodic Alumina. *Nanotechnology* **2007**, *18*, 365601, doi:10.1088/0957-4484/18/36/365601.
9. Shang, G.L.; Fei, G.T.; Zhang, Y.; Yan, P.; Xu, S.H.; Zhang, L.D. Preparation of Narrow Photonic Bandgaps Located in the near Infrared Region and Their Applications in Ethanol Gas Sensing. *J. Mater. Chem. C* **2013**, *1*, 5285–5291, doi:10.1039/C3TC30782D.
10. Yan, P.; Fei, G.-T.; Li, H.; Shang, G.; Wu, B.; Zhang, L. Alumina Photonic Crystals with Defect Modes for Sensor Application. *Chin. J. Chem. Phys.* **2014**, *27*, 121–124, doi:10.1063/1674-0068/27/01/121-124.
11. Yan, P.; Fei, G.T.; Shang, G.L.; Wu, B.; Zhang, L.D. Fabrication of One-Dimensional Alumina Photonic Crystals with a Narrow Band Gap and Their Application to High-Sensitivity Sensors. *J. Mater. Chem. C* **2013**, *1*, 1659–1664, doi:10.1039/C2TC00396A.
12. Born, M.; Wolf, E. *Principles of Optics: Electromagnetic Theory of Propagation, Interference and Diffraction of Light*; 7th ed.; Cambridge University Press: Cambridge, 2013; ISBN 978-1-139-64418-1.
